# Complete Genome Analysis and Antimicrobial Mechanism of *Burkholderia gladioli* ZBSF BH07 Reveal Its Dual Role in the Biocontrol of Grapevine Diseases and Growth Promotion in Grapevines

**DOI:** 10.3390/microorganisms13081756

**Published:** 2025-07-28

**Authors:** Xiangtian Yin, Chundong Wang, Lifang Yuan, Yanfeng Wei, Tinggang Li, Qibao Liu, Xing Han, Xinying Wu, Chaoping Wang, Xilong Jiang

**Affiliations:** 1 Shandong Academy of Grape, Shandong Academy of Agricultural Sciences, Jinan 250100, China; yxt1985@163.com (X.Y.); wangchundong@saas.ac.cn (C.W.); yuanlifang@saas.ac.cn (L.Y.); weiyanfeng@saas.ac.cn (Y.W.); weifengluolu@126.com (T.L.); liuqibao@saas.ac.cn (Q.L.); hanxing@nwafu.edu.cn (X.H.); wuxinying@saas.ac.cn (X.W.); wangchaoping@saas.ac.cn (C.W.); 2 Shandong Key Laboratory for Green Prevention and Control of Agricultural Pests, Shandong Academy of Agricultural Sciences, Jinan 250100, China

**Keywords:** *Burkholderia gladioli*, grape, antimicrobial mechanism, growth promoting, comparative genome analysis, secondary metabolites

## Abstract

*Burkholderia gladioli* is a multifaceted bacterium with both pathogenic and beneficial strains, and nonpathogenic *Burkholderia* species have shown potential as plant growth-promoting rhizobacteria (PGPRs) and biocontrol agents. However, the molecular mechanisms underlying their beneficial functions remain poorly characterized. This study systematically investigated the antimicrobial mechanisms and plant growth-promoting properties of *B. gladioli* strain ZBSF BH07, isolated from the grape rhizosphere, by combining genomic and functional analyses, including whole-genome sequencing, gene annotation, phylogenetic and comparative genomics, in vitro antifungal assays, and plant growth promotion evaluations. The results showed that ZBSF BH07 exhibited broad-spectrum antifungal activity, inhibiting 14 grape pathogens with an average inhibition rate of 56.58% and showing dual preventive/curative effects against grape white rot, while also significantly promoting grape seedling growth with increases of 54.9% in plant height, 172.9% in root fresh weight, and 231.34% in root dry weight. Genomic analysis revealed an 8.56-Mb genome (two chromosomes and one plasmid) encoding 7431 genes and 26 secondary metabolite biosynthesis clusters (predominantly nonribosomal peptide synthetases), supporting its capacity for antifungal metabolite secretion, and functional analysis confirmed genes for indole-3-acetic acid (IAA) synthesis, phosphate solubilization, and siderophore production. These results demonstrate that ZBSF BH07 suppresses pathogens via antifungal metabolites and enhances grape growth through phytohormone regulation and nutrient acquisition, providing novel insights into the dual mechanisms of *B. gladioli* as a biocontrol and growth-promoting agent and laying a scientific foundation for developing sustainable grapevine disease management strategies.

## 1. Introduction

Owing to their crucial roles in reducing dependence on agrochemicals, enhancing crop stress tolerance, and advancing sustainable agriculture, plant growth-promoting rhizobacteria (PGPR) have gained significant international recognition in agricultural research [1,2,3]. The genus *Burkholderia*, which comprises functionally diverse gram-negative bacteria, was first described by Walter Burkholder in 1942 and was established as a distinct taxon by Yabuuchi et al. in 1992 [4,5]. To date, this genus encompasses 127 validly published species [6]. Members of *Burkholderia* are widely distributed in soil, aquatic environments, and plant/animal tissues and are broadly categorized into pathogenic and nonpathogenic groups. Notably, nonpathogenic *Burkholderia* strains such as *B. phytofirmans* PsJN, *B. ambifaria* XN08, and *B. gladioli* KRS027 serve as vital PGPR agents, demonstrating exceptional root colonization capabilities, prolific secondary metabolite synthesis, and synergistic effects in biocontrol and plant growth promotion through mechanisms such as nutrient solubilization, induced systemic resistance, and pathogen suppression [7,8,9]. Conversely, species within this genus are known pathogens that affect animals, humans, and plants, including *B. cenocepacia*, *B. gladioli* BSR3, and *B. glumae* BGR1 [10,11,12]. These pathogenic strains cause host damage through toxin production, tissue destruction, or immune response dysregulation [13]. Despite its pathogenic member species, the overall diversity and functional value of the *Burkholderia* genus remain substantial. Nonpathogenic *Burkholderia* strains exhibit particularly promising applications in agriculture and environmental protection. In addition to promoting plant growth, they play critical roles in biocontrol, providing robust support for sustainable agricultural development.

In the field of biological control, the antimicrobial activity of *Burkholderia* species primarily stems from their extensive repertoire of secondary metabolites. Genomic analyses revealed that strains within this genus typically harbor 25–35 biosynthesis-related gene clusters (BGCs) on average, encompassing pathways responsible for synthesizing nonribosomal peptides (NRPS), polyketides (PKS), siderophores, and other compounds [14]. Notably, several *Burkholderia* strains exhibit broad-spectrum antagonism against major phytopathogens, including *Verticillium dahliae*, *Sporisorium scitamineum*, *Magnaporthe oryzae*, *Fusarium oxysporum*, and *Botrytis cinerea* [9,15,16,17]. This activity is exemplified by specific antimicrobial agents: AFC-BC11, produced by *B. cepacia* BC11, effectively suppresses soil-borne pathogenic fungi [18], whereas *B. gladioli* BCC0238 produces gladiolin, a novel macrolide antibiotic that has potent activity against *Mycobacterium tuberculosis* H37Rv [19]. Another study on *B. gladioli* strain NGJ1 identified a glycosyltransferase-like toxin as a potent antifungal protein, which exhibits potential for the control of sheath blight disease in rice by targeting fungal pathogens [20].

With respect to plant growth promotion, substantial evidence confirms the potential of *Burkholderia* strains [21,22,23] to enhance plant development through multiple mechanisms, such as the production of phytohormones (e.g., indole-3-acetic acid (IAA), cytokinins, and gibberellins) and improved nutrient acquisition via nitrogen fixation and phosphate solubilization. For example, *B. cepacia* SCAUK0330 solubilizes phosphate at concentrations exceeding 450 μg/mL, promoting growth in both healthy maize plants and those infected by *Helminthosporium maydis* [21]. *B. anthina* MYSP113 significantly increases sugarcane fresh weight, dry weight, and chlorophyll content at 45 and 90 days after inoculation [24]. *B. gladioli* BA-7 enhances biomass and leaf nutrient concentrations in 1103P (*Vitis berlandieri* × *Vitis rupestris*) grape rootstocks [25]. Inoculation of *Vitis vinifera* L. cv. Chardonnay explants with the PGPR strain *B. phytofirmans* PsJN improved growth and physiological activity under low-temperature stress [7].

Despite these advances, critical knowledge gaps remain: The molecular mechanisms underlying the dual biocontrol and plant growth-promoting traits of *Burkholderia* strains are not fully elucidated, particularly regarding the genomic basis of secondary metabolite biosynthesis and the regulatory networks linking nutrient acquisition to plant growth promotion. Furthermore, systematic studies integrating genomic analysis with functional validation of beneficial traits are still limited for grape-associated *Burkholderia* strains, restricting the development of targeted biocontrol and growth-promotion strategies for sustainable viticulture. This present study has focused on a new *Burkholderia* sp. isolated from the grape rhizosphere and designated *Burkholderia* sp. strain ZBSF BH07, aiming to elucidate the molecular mechanisms underlying both its antimicrobial activity and its plant growth promotion through whole-genome analysis. The antagonistic activity of ZBSF BH07 against 14 fungal pathogens, including *Coniella vitis*, has been demonstrated along with its growth-promoting effects on grapevines and its capacity to increase grape growth through IAA production and phosphate solubilization. Genome sequencing and functional annotation of the strain have revealed the evolutionary characteristics of key functional genes via comparative genomics. This work provides new scientific foundations for developing environmentally sustainable strategies to control grapevine diseases.

## 2. Materials and Methods

### 2.1. Strains, Culture Conditions, and Plant Material

*Coniella vitis*, *Botrytis cinerea*, *Botryosphaeria dothidea*, *Pestalotiopsis clavispora*, *Diaporthe eres*, *Fusarium oxysporum*, *Aspergillus niger*, *Fusarium graminearum*, *Fusarium pseudograminearum*, *Colletotrichum aenigma*, *C. siamense*, *C. viniferum*, *C. gloeosporioides*, and *C. fructicola* strains were cultured on potato dextrose agar (PDA) media (200 g/L; glucose, 20 g/L; agar, 15 g/L) at 25 °C. Annual grape (*Vitis vinifera* cv. Red Globe) seedlings were grown in 14 cm diameter and 17 cm tall pots, with a seedling substrate prepared by mixing nutrient soil and vermiculite at a 1:1 volume ratio. The plants were watered once weekly and maintained in a 25 °C environment with a 16 h light/8 h dark photoperiod [26].

### 2.2. Isolation of Strains and In Vitro Antifungal Activity Assays

*B. gladioli* ZBSF BH07 was isolated from healthy grape rhizosphere soil in Shandong Province, China, in May 2023. The isolation procedure was as follows: 10 g of soil was weighed and added to an Erlenmeyer flask containing 90 mL of sterile physiological saline, followed by shaking culture at 180 r/min for 1 h. Strains were isolated using the dilution plate method, and after colony growth, single colonies were picked and streaked on Luria Bertani (LB) solid medium (10 g/L tryptone, 5 g/L NaCl, and 5 g/L yeast extract, agar, 15 g/L) at 28 °C [27]. Using *C. vitis* as the indicator strain, the confrontation culture method was employed to screen for strains with significant antagonistic activity against the target fungus, and the hyphal morphology on the confrontation plates was observed using an optical microscope (Optika B-383PL, Ponteranica, Italy). The strain ZBSF BH07 was selected and deposited in the China General Microbiological Culture Collection Center (strain number 30228).

The in vitro antifungal activity of ZBSF BH07 against 14 pathogenic fungi was assessed via a confrontation culture assay. A 5 mm diameter mycelial disc, excised from a 5-day-old pathogenic fungus, was inoculated at the center of a PDA plate. Additionally, 2 μL of ZBSF BH07 bacterial suspension (OD_600_ = 1.0) was inoculated at 30 mm from the center along the perimeter of the plate. LB broth was used as the control. The plates were incubated at 25 °C for 5 days [28]. Each treatment had at least three biological replicates, and the assay was replicated twice. The percentage of growth inhibition was calculated via the following equation: n = [(A − B)/A] × 100, where A is the colony diameter of the control fungus and B is the colony diameter of the treated fungus.

### 2.3. Whole-Genome Sequencing and Assembly

The genomic DNA of *B. gladioli* ZBSF BH07 was sequenced at Biomarker Technologies via the Pacific Biosciences (PacBio, Menlo Park, CA, USA) RSII single-molecule real-time (SMRT) sequencing platform [29]. For genome assembly, filtered subreads were first assembled with Canu v1.5 software, followed by genome circularization via Circlator v1.5.5 [30]. Concurrently, a 10 kb insert-size template library was prepared according to the PacBio Sequel gDNA protocol and subjected to sequencing on the PacBio Sequel instrument. Finally, circular genome alignment visualization was performed via Circos v0.66 [31].

### 2.4. Gene Prediction and Functional Annotation

Coding gene prediction was performed via Prodigal v2.6.3 [32]. The GenBlastA v1.0.4 program was used to scan the whole genomes after masking the predicted functional genes. Putative candidates were then analyzed by searching for non-mature mutations and frameshift mutations via GeneWise v2.2.0. Transfer RNA (tRNA) genes were predicted with tRNAscan-SE v2.0 [33], and ribosome RNA (rRNA) genes were predicted with Infernal v1.1.3. Repetitive sequences were predicted via RepeatMasker. PhiSpy v2.3 was used for prophage prediction, and CRT v1.2 was used for CRISPR identification. IslandPath-DIMOB v0.2 was used to predict genomic islands in the genome. and PromPredict v1 was used for promoter prediction. For functional annotation, the predicted proteins were blasted (e-value: 1 × 10^−5^) against Nr, Swiss-Prot, TrEMBL, KEGG, and eggnog, and Blast2GO 6.0 was subsequently used for GO annotation. Furthermore, pathogenicity and drug resistance can be researched via BLAST (https://blast.ncbi.nlm.nih.gov/Blast.cgi, accessed on 10 January 2025) searches against the CAZy, TCDB, CARD, and PHI databases.

### 2.5. Phylogenetic Tree Construction

The evolutionary position of *Burkholderia gladioli* ZBSF BH07 was determined via 16S rDNA gene sequence analysis and the multilocus sequence analysis (MLSA) method [34,35,36]. A total of 32 *Burkholderia* strains were selected for phylogenetic tree construction to investigate the evolutionary relationships of strain ZBSF BH07 (Table S1). For MLSA, five housekeeping genes (16S rRNA, *gyrB*, *atpD*, *trpB*, and *rpoD*) were selected. Sequence alignment of ZBSF BH07 with other *Burkholderia* strains was conducted via the maximum likelihood clustering method implemented in MEGA7 with 1000 bootstrap replicates to generate phylogenetic trees.

### 2.6. Comparative Genomics Analysis and Mining for Genes Related to Plant-Beneficial Traits

For comparative genomic analysis, the genome sequences of *B. gladioli* ZBSF BH07 were compared with those of *B. gladioli* CGB10, *B. gladioli* KRS027, *B. gladioli* BK04, *B. gladioli* BBB01, and *B. gladioli* BSR3 via MAUVE comparison software 2.4.0 [37]. Additionally, a circular chromosomal map of all the genomes used in the pangenome analysis was prepared via BLAST Ring Image Generator (BRIG) v 0.95, with strain ZBSF BH07 used as a reference genome [38]. Furthermore, average nucleotide identity (ANI) was determined via the orthologous average nucleotide identity (OrthoANI) tool (http://www.ezbiocloud.net/sw/oat, accessed on 20 May 2025), and in silico DNA–DNA hybridization (DDH) was performed via the Genome-to-Genome Distance Calculator (GGDC) 3.0 from the website https://ggdc.dsmz.de/ggdc.php# (accessed on 20 May 2025): on the basis of BLAST+ 2.14.0 alignment, the DDH value was estimated via the calculation of high-similarity fragments between genomes [39]. Functional genes involved in plant growth promotion, such as genes responsible for IAA production, phosphate solubilization, chemotaxis, and the assembly of flagella, were searched in NCBI databases as described by Kumar et al. [40]. A BLAST search was performed against the locally constructed database of the publicly available genomes of *B. gladioli*, with the genome of *B. gladioli* ZBSF BH07 as a query. The identities of different functional genes at the nucleotide level were compared among the strains via BLAST [40]. The secondary metabolite gene clusters were predicted via antiSMASH 8.0, with “high” for a cluster similarity of greater than or equal to 75%, “medium” for a cluster similarity between 75% and 50%, and “low” for a cluster similarity between 15% and 50% [41].

### 2.7. Morphological, Physiological, and Biochemical Tests

Strain ZBSF BH07 was cultured on LB media plates and incubated at 28 °C for 48 h to observe colony characteristics, such as color, morphology, and growth properties. The morphology of the strain was observed by scanning electron microscopy (SEM, Hitachi, SU8600, Tokyo, Japan) and transmission electron microscopy (TEM, Hitachi HT7800, Tokyo, Japan). The growth curve and the dynamic change in pH were measured every 4 h via a spectrophotometer (Persee, TU-1900, Beijing, China) and pH meter (Sartorius, PB-10, Göttingen, Germany). The hemolysis of ZBSF BH07 was evaluated by culturing the strains on Columbia blood plates at 28 °C for 48 h as described previously [26].

### 2.8. Measurement of Siderophore Production and Phosphate Solubilization

Organophosphorus media (10.0 g/L glucose, 0.5 g/L [NH_4_]_2_SO_4_, 0.3 g/L NaCl, 0.3 g/L MgSO_4_, 0.03 g/L MnSO_4_, 0.3 g/L KCl, 0.03 g/L FeSO_4_, 0.2 g/L lecithin, 1.0 g/L CaCO_3_, 15.0 g/L agar, pH 7.4) supplemented with insoluble lecithin was used to assess organophosphate solubilization by ZBSF BH07. Pikovskaya (PVK) medium (10.0 g/L glucose, 0.5 g/L [NH_4_]_2_SO_4_, 0.3 g/L NaCl, 0.3 g/L MgSO_4_, 0.03 g/L MnSO_4_, 0.3 g/L KCl, 0.03 g/L FeSO_4_, 5.0 g/L Ca_3_(PO_4_)_2_, and 15.0 g/L agar, pH 7.4) with insoluble Ca_3_(PO_4_)_2_ was used to test the ability of ZBSF BH07 to solubilize inorganic phosphorus. Siderophore production by ZBSF BH07 was determined via blue agar media containing chrome azurol S (CAS), hexadecyltrimethylammonium bromide (CTAB), and iron ions. The CAS agar plate gives a qualitative assessment of siderophore production through the appearance of yellow circles surrounding colonies for which siderophore concentration is increasing [9].

### 2.9. Assessment of Biocontrol Activity and Plant Growth Promotion

For the biocontrol assay of detached grape berries and leaves, strain ZBSF BH07 was applied via the mycelial plug inoculation method to inoculate healthy detached Red Globe grape berries and leaves. The pathogenic strain *C. vitis* GP1 was first cultured on PDA plates at 25 °C for 3 days. Healthy grape berries were rinsed three times with sterile water and surface sterilized, and then each fruit was punctured once a 0.5 mm diameter sterile insect pin was used to inoculate a 5 mm mycelial disc. For the control groups, PDA plugs without fungal mycelia and sterile water were used as parallel controls. To evaluate the curative effect, ZBSF BH07 cultures at a concentration of 10^8^ CFU/mL were sprayed onto the inoculated fruits and leaves 1 day after pathogenic inoculation. Additionally, to assess preventive efficacy, ZBSF BH07 cultures were sprayed prior to inoculation with the pathogenic strain *C. vitis* GP1. All treated leaves and berries were incubated at 28 °C and 90% relative humidity (RH). Five days postinoculation, the lesion diameters on leaves and fruits, as well as the biocontrol efficiency of ZBSF BH07 cultures, were recorded [27,28].

One-year-old grape seedlings were used to evaluate the plant growth-promoting function of strain ZBSF BH07 after they developed three true leaves. The preparation of the ZBSF BH07 cell suspension followed these steps: ZBSF BH07 was first inoculated into LB broth and cultured with shaking at 28 °C for 24 h. The cells were then collected by centrifugation at 8000 rpm, washed with ddH_2_O, and resuspended to adjust the optical density to an OD_600_ value of 1.0. The prepared ZBSF BH07 suspension was applied to the roots of the grape plants at a volume of 200 mL per pot. This inoculation procedure was repeated 7 days after the initial application. Thirty days following the third inoculation, the length and weight of the shoots and roots were measured. Each experimental group included at least six seedlings, and all measurements were performed with three biological replicates.

### 2.10. Statistical Analysis

The data were analyzed via ANOVA and Duncan’s multiple range test (*p* ≤ 0.05) via the statistical software SPSS version 19.0 (SPSS Inc., Chicago, IL, USA). The significance between the means of different treatments was evaluated via Duncan’s test (D). test.

## 3. Results

### 3.1. Biocontrol Effect of Isolate ZBSF BH07 Against Plant Pathogens

**Figure 1:**
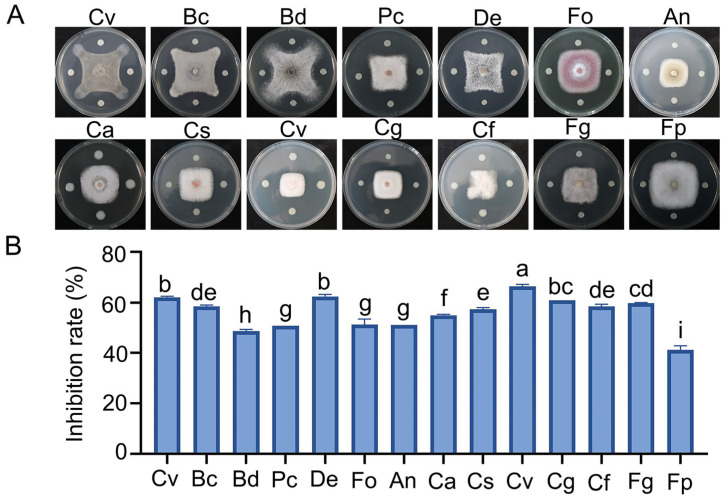
Antagonistic activity of *Burkholderia gladioli* ZBSF BH07 against pathogenic fungi. (**A**) Antagonistic assay of *B. gladioli* ZBSF BH07. *Coniella vitis* (Cv), *Botrytis cinerea* (Bc), *Botryosphaeria dothidea* (Bd), *Pestalotiopsis clavispora* (Pc), *Diaporthe eres* (De), *Fusarium oxysporum* (Fo), *Aspergillus niger* (An), *Colletotrichum aenigma* (Ca), *C. siamense* (Cs), *C. viniferum* (Cv), *C. gloeosporioides* (Cg), *C. fructicola* (Cf), *Fusarium graminearum* (Fg), and *Fusarium pseudograminearum* (Fp). (**B**) Inhibition rate of each microorganism. Data are the mean ± SE (three experiments). Different letters indicate significant differences (*p* < 0.05) according to Tukey’s test.

Ninety strains were isolated from the rhizosphere soil of vineyards, among which ZBSF BH07 exhibited the strongest antifungal activity against *C. vitis*, with a mycelial growth inhibition rate of 63.45% (Figure S1A,B). Microscopic observation revealed severe morphological abnormalities in *C. vitis* hyphae under antagonistic culture conditions compared with the control group, including hyphal thinning, fragmentation, rupture, and cytoplasmic leakage (Figure S1C,D). Confrontation culture assays demonstrated that strain ZBSF BH07 exhibited varying degrees of inhibitory effects against all 14 fungal strains, with an average inhibition rate of 56.58%. Among these, the most significant inhibitory effect was observed against *C. viniferum*, reaching 66.39%. This was followed by inhibition rates of 62.1%, 62.41%, and 60.9% against *C. vitis*, *D. eres*, and *C. gloeosporioides*, respectively. In contrast, relatively weaker inhibitory effects were observed against *F. pseudograminearum* and *B. dothidea*, at 41.20% and 48.7%, respectively. The inhibition rates of other pathogenic fungi ranged from 50.8% to 59.7% (Figure 1A,B). These results indicate that ZBSF BH07 can secrete antifungal metabolites to exert inhibitory effects and that it possesses broad-spectrum antifungal activity.

### 3.2. Efficacy of ZBSF BH07 in Controlling Grape White Rot

**Figure 2:**
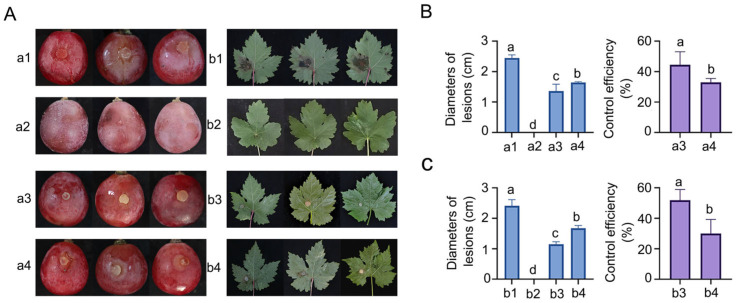
Biocontrol efficiency of *B. gladioli* ZBSF BH07 against grape white rot. (**A**) (**a1**,**b1**) Inoculation with *C. vitis*; (**a2**,**b2**) culture of ZBSF BH07; (**a3**,**b3**) inoculated with *C. vitis* 24 h after inoculation with the culture of ZBSF BH07; (**a4**,**b4**) inoculated culture of ZBSF BH07 24 h after inoculation with *C. vitis*. Lesion diameters and control efficiency of *B. gladioli* ZBSF BH07 on grape berries (**B**) and leaves (**C**). Letters (a, b, c, and d) above the bars indicate significant differences at *p* < 0.05 according to Duncan’s multiple range test.

This study evaluated the control efficacy of the ZBSF BH07 strain against *C. vitis* through in vitro assays. The experimental results indicated that strain ZBSF BH07 exerted dual preventive and curative effects against grape white rot, with preventive efficacy significantly outperforming curative efficacy. In grape leaf assays, preventive application of fermentation broth (fermentation broth pretreatment) demonstrated marked control efficiency: lesion expansion was constrained to 1.15 mm (a 52.28% reduction compared with the control group), achieving a control efficiency of 51.91%. In contrast, curative application following pathogen infection resulted in a lower control efficiency of 30.05%, with lesion expansion measuring 1.67 mm (Figure 2A,C). In the grape berry assays, the preventive treatment group presented a lesion diameter of 1.36 mm, representing a 44.45% reduction relative to the control. Notably, its control efficiency (44.45%) was significantly greater than that of the curative treatment group, which was 32.90% with a lesion diameter of 1.64 mm (Figure 2A,B). Importantly, inoculation of ZBSF BH07 alone did not induce toxicity in detached grape berries or leaves.

### 3.3. Effects of ZBSF BH07 on Plant Growth Promotion

**Figure 3:**
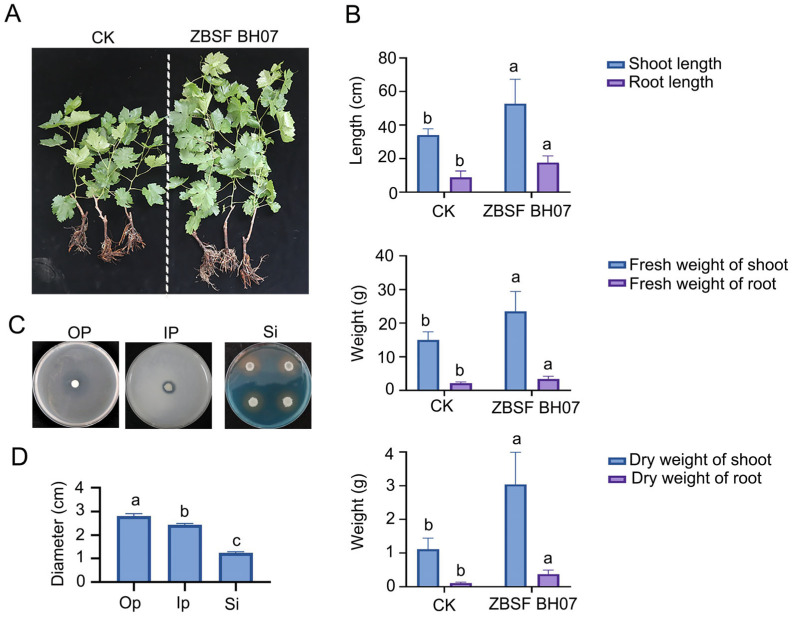
Growth-promoting effect of *B. gladioli* ZBSF BH07 on grape. (**A**) Phenotypic comparison of plants treated with CK (control) and *B. gladioli* ZBSF BHO7. (**B**) Bar charts showing the biomass of shoots and roots of plants treated with CK or ZBSF BHO7. (**C**) PGP assays for solubilization of organophosphorus (Op) and inorganic phosphorus (Ip), and siderophore (Si) production by ZBSF BH07. (**D**) Diameter of the phosphate solubilization zones (Op or Ip) and siderophore production (Si) zones observed on PGP assay plates growing ZBSF BH07. Letters (a, b, and c) above the bars indicate significant differences at *p* < 0.05 according to Duncan’s multiple range test.

The results of the growth-promoting experiments of ZBSF BH07 on grape seedlings demonstrated its significant promotional effect on plant growth. Compared with those in the control group, the shoot growth indicators in the treatment group improved as follows: plant height increased by 18.67 cm (a 54.9% increase), shoot fresh weight increased by 8.483 g (a 56.51% increase), and shoot dry weight increased by 1.207 g (a 55.74% increase) (Figure 3A,B). Strikingly, the root growth indicators exhibited even more pronounced enhancements: the root length increased by 8.75 cm (a 100.0% increase), the root fresh weight increased by 1.925 g (a 172.9% increase), and the root dry weight increased by 0.2583 g (a 231.34% increase) (Figure 3A,B). These findings indicate that the ZBSF BH07 treatment not only significantly promoted the growth of aboveground plant parts but also more effectively stimulated root system development.

Assays evaluating plant growth-promoting (PGP) traits, including siderophore production and phosphate solubilization, revealed the following results. On blue chrome azurol S (CAS) plates, ZBSF BH07 colonies were surrounded by orange–yellow halos with a diameter of 2.5 cm, indicating their capacity to produce siderophores. On organic phosphorus and inorganic phosphorus plates, clear zones with diameters of 2.8 cm and 1.5 cm were observed, respectively (Figure 3C,D), demonstrating ZBSF BH07’s ability to solubilize lecithin and tricalcium phosphate.

### 3.4. General Genomic Features of B. gladioli ZBSF BH07

**Figure 4:**
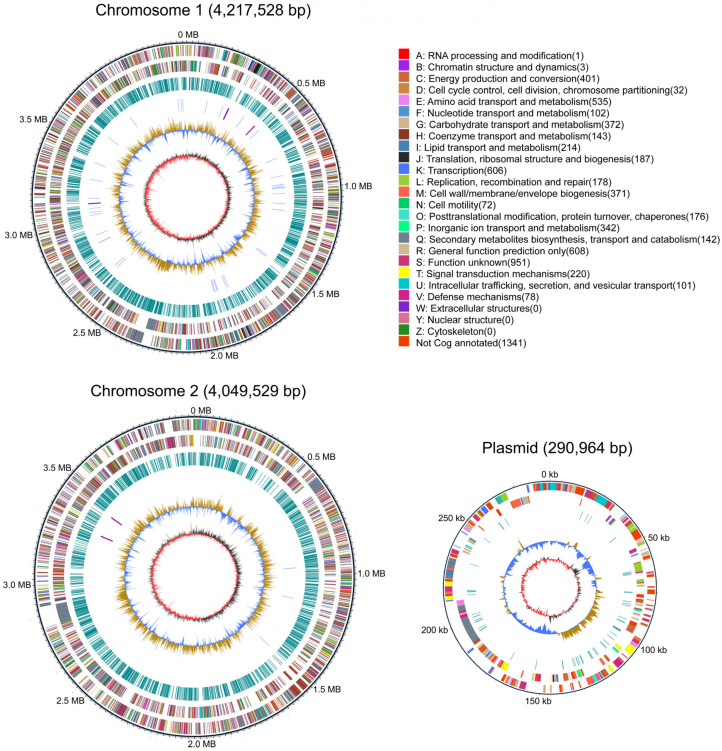
Genome map of *B*. *gladioli* ZBSF BH07. The distribution of the circle from the outside indicates the genome size, forward CDS, reverse CDS, repeat sequence, tRNA (blue), rRNA (purple), GC ratio (yellow and blue indicate regions where the GC ratio is higher than average and lower than average, respectively), and CG skew positive (dark) and negative (red).

The completed genome of *B. gladioli* ZBSF BH07 has been shown to be composed of three contigs (contiguous overlapping DNA fragments) assembled into two circular chromosomes (Chr1 and Chr2) and one plasmid (Figure 4). Chr1 contained 4,217,528 bp with 67% GC content, Chr 2 had 4,049,529 bp with 68% GC content, and the plasmid comprised 290,964 bp with 64% GC content. The details of the assembly information and genomic features are summarized in Table S2. A total of 7431 predicted genes were identified in the genome, including 7262 protein-coding sequences, 15 ribosomal RNAs, and 68 tRNAs (Table S2). The genes associated with transcription (8.16%) had the highest density, followed by amino acid transport and metabolism (7.20%), energy production and conversion (5.40%), carbohydrate transport and metabolism (5.01%), cell wall/membrane/envelope biogenesis (4.99%), inorganic ion transport and metabolism (4.60%), lipid transport and metabolism (2.88%), and translation, ribosomal structure, and biogenesis (2.52%). In addition, 25 CRISPRs were involved in ZBSF BH07, and the length of the repeated sequences ranged from 19 to 38 bp (Table S3).

### 3.5. Morphological Identification and Phylogenetic Analysis of B. gladioli ZBSF BH07

**Figure 5:**
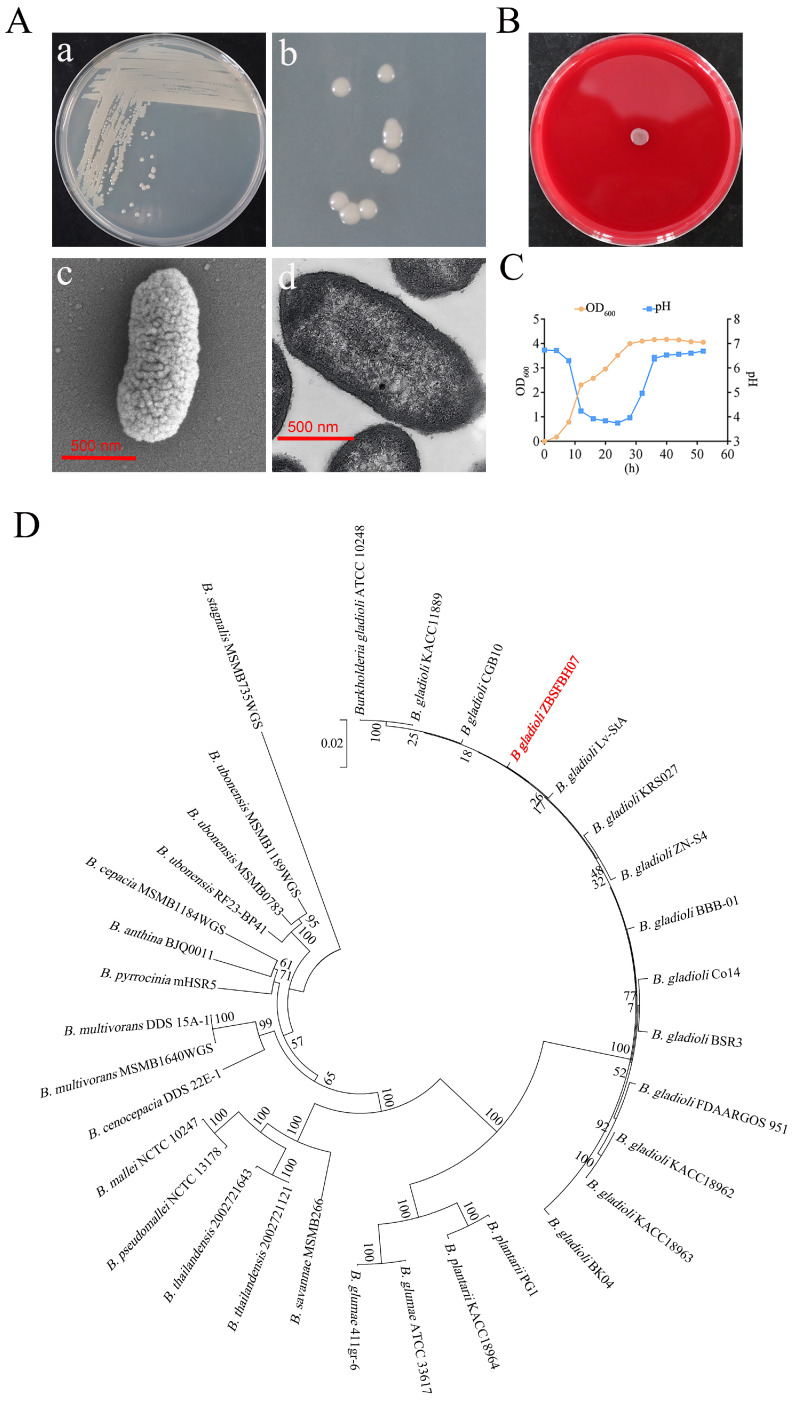
General characteristics of *B. gladioli* ZBSF BH07. (**A**) Morphological identification of ZBSF BH07. (**a**,**b**) Colony morphology. Cell structure under scanning (**c**) and transmission (**d**) electron microscopy. (**B**) Hemolysis test using Columbia blood plates. (**C**) Growth dynamics and pH changes in ZBSF BH07. (**D**) Phylogenetic tree based on five housekeeping genes (16S rRNA, *gyrB*, *atpD*, *trpB*, *rpoD*), showing the relationship between ZBSF BH07 (in red) relationship and other *Burkholderia* species (bootstrap values, 1000 replicates; scale bar = 0.05 substitutions per nucleotide).

After 48 h of streak-culturing on LB agar plates, single colonies of ZBSF BH07 exhibited grayish–white coloration with a smooth, glossy surface and regular margins (Figure 5A). Scanning electron microscopy (SEM) and transmission electron microscopy (TEM) revealed that ZBSF BH07 is a rod-shaped bacterium, with a length ranging from 1.1 to 1.8 μm and a width ranging from 0.4 to 0.6 μm, and it possesses flagella (Figure 5A). A blood agar hemolysis assay confirmed that strain ZBSF BH07 was unable to produce hemolysin activity on plates (Figure 5B). The growth curve analysis of the strain revealed distinct physiological phases during the 52 h cultivation period. Following an initial adaptation phase, the culture entered exponential growth at 8 h postinoculation, with the optical density (OD_600_) increasing from 0.18 to 4.00 during the 8–28 h period. The stationary phase commenced at 40 h, when it peaked at OD_600_ of 4.17, followed by gradual culture autolysis. Concurrent pH monitoring revealed dynamic environmental changes: medium acidification was initiated at 8 h (pH 6.3), reaching minimum acidity (pH 3.75) at 24 h. The pH subsequently recovered to 6.69 by 52 h (Figure 5C).

Phylogenetic analysis was performed for the identification of strain ZBSF BH07. The phylogenetic tree constructed on the basis of 16S rRNA gene sequences indicated that ZBSF BH07 is closely related to *B. gladioli* strains (Figure S2). Additionally, the phylogenetic tree generated via multilocus sequence analysis (MLSA) with concatenated genes, including 16S rRNA, *gyrB*, *atpD*, *trpB*, and *rpoD*, further confirmed that ZBSF BH07 is most closely related to *B. gladioli* CGB10 and *B. gladioli* LvStA (Figure 5D).

### 3.6. Comparison of the B. gladioli ZBSF BH07 Genome with Those of Other Completely Sequenced B. gladioli Strains

#### 3.6.1. Comparison of ZBSF BH07 with the Genomic Information of Other *B. gladioli*

The complete genome sizes of the six *B. gladioli* strains ranged from 8.04 to 9.05 megabases (Mb), with the GC content varying between 67.40% and 68.67% and the number of predicted coding genes ranging from 6768 to 8781. Additionally, all six strains contain two chromosomes and 0 to 4 plasmids: *B. gladioli* BSR3 harbors 4 plasmids, *B. gladioli* BK04 contains no plasmids, and the remaining strains each have two plasmids. Additional genomic features of the six strains are detailed in Table 1.

**Figure 6:**
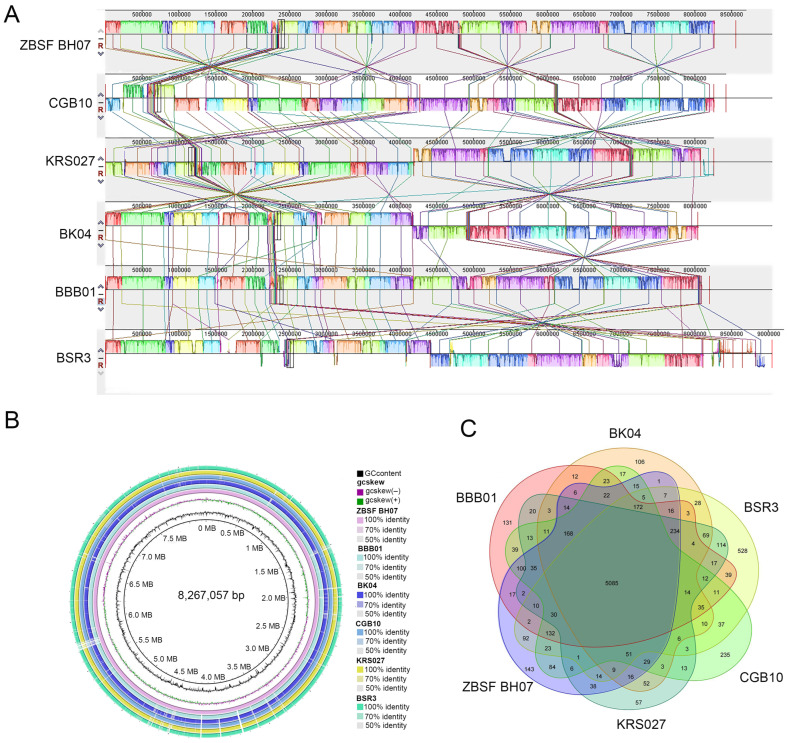
Genomic comparison of *B. gladioli* ZBSF BH07 with five closely related strains. (**A**) Synteny analysis (MAUVE) showing conserved (colored boxes) and inverted (boxes below the line) genomic regions. (**B**) Pangenome analysis (excluding plasmids) highlighting unique genes in ZBSF BH07 (outermost circle). (**C**) Venn diagram of shared and unique orthologous gene clusters.

To evaluate the evolutionary distances between these sequenced strains and several *B. gladioli* strains, the genome sequence of ZBSF BH07 was aligned with five sequenced *B. gladioli* strains (CGB10, KRS027, BK04, BBB01, and BSR3) via Mauve software. Comparative analysis revealed significant horizontal gene transfer (HGT) events among *B. gladioli* strains. Furthermore, the ZBSF BH07 genome presented extensive regions with conserved sequences and gene orders relative to those of other *B. gladioli* strains, with the highest similarity to CGB10 (Figure 6A). To identify conserved and strain-specific regions at the nucleotide level, this study used the BRIG to align the genomes of the six strains, with *B. gladioli* ZBSF BH07 as the reference genome (Figure 6B). The circular image illustrates the comparative results of the six *B. gladioli* genomes, confirming close genomic associations between ZBSF BH07 and other *B. gladioli* strains.

Gene family analysis revealed that these six sequenced *B. gladioli* strains share 5085 core genes. Specifically, ZBSF BH07 shares 6351, 6045, 5883, 5742, and 5850 genes with CGB10, BBB01, BSR3, KRS027, and BK04, respectively. ZBSF BH07 harbored 544, 534, 696, 729, and 837 unique genes relative to CGB10, BBB01, BSR3, BK04, and KRS027, respectively. Notably, the *B. gladioli* ZBSF BH07 genome contains 143 strain-specific genes (Figure 6C).

#### 3.6.2. ANI and DDH Analysis

ANI analysis revealed that pairwise ANI values among the six genomes ranged from 97.82% to 99.27%. Specifically, the ANI values between ZBSF BH07 and *B. gladioli* CGB10, BSR3, BBB01, BK04, and KRS027 were 99.27%, 98.00%, 99.06%, 98.75%, and 98.66%, respectively (Figure S3). The pairwise DDH values among the six genomes ranged from 80.9% to 93.7%. The highest DDH value was detected between CGB10 and ZBSF BH07 (93.7%), whereas the lowest was observed between BSR3 and KRS027 (80.9%) (Figure S3). Notably, all DDH values among these genomes were relatively high (>80%), indicating strong genetic similarity between the strains.

#### 3.6.3. Genetic Basis for Promoting Plant Growth

Through systematic analysis of plant growth-promoting genes in *Burkholderia gladioli* ZBSF BH07 and closely related strains, this study revealed both high conservation and specific variations in the phosphate solubilization and IAA biosynthesis pathways. In the phosphate metabolism system, core functional genes exhibited significant evolutionary stability: the *pstS* and *pstB* genes maintained average homologies of 99.82% (range 99.42–100%) and 99.86% (range 99.29–100%), respectively, with the *phoU* gene showing complete sequence conservation (100%) across all compared strains. The *pqqA* gene, a key component of the pyrroloquinoline quinone (PQQ) synthesis pathway, demonstrated absolute genetic stability with 100% homology among all strains. Notably, the BK04 strain displayed specific genetic variations: its *pstB* gene homology (99.29%) was significantly lower than that in other strains (100% in CGB10, KRS027, BBB01, and BSR3), and its *phnA* (phosphonoacetate hydrolase) gene homology (98.40%) was also below the average level of other strains (99.20%). In the IAA biosynthesis pathway, the core tryptophan synthesis genes *trpD*/*trpE* maintained high conservation (>99.50% homology). However, the *trpA* gene homology decreased to 98.03% in the BSR3 strain. Further analysis revealed significant sequence divergence in the *aroA* gene (95.39% homology) in BSR3, which is involved in aromatic amino acid synthesis. Of particular note was the systematic absence (annotated as “NA”) of *iaaH* (indoleacetamide hydrolase) and aroA genes in KRS027, BK04, and BBB01 strains, suggesting that genomic structural variations may compromise the integrity of the IAA biosynthesis pathway in these strains (Table 2).

Flagellar gene homology showed variation across the five strains (99.60% in CGB10 to 98.12% in BSR3). Multiple genes (*cheY*, *flgB*, *flgC*, etc.) maintained 100% homology in all strains, while fliL showed the lowest homology (75.76%) in BSR3, indicating potential structural divergence. Chemotaxis-related genes were generally highly conserved, with most showing >98% homology across strains (Table S4).

### 3.7. Genes/Gene Cluster for Antibiotic Synthesis

To characterize the genome of ZBSF BH07 comprehensively and classify its potential antagonistic genes, this study employed antiSMASH v8.0 software to predict and analyze secondary metabolite BGCs. The results revealed that the ZBSF BH07 strain harbors a total of 26 functional BGCs, which are specifically distributed as follows: 8 BGCs identified on chromosome 1, 17 on chromosome 2, and 1 on the plasmid. Further analysis revealed that nonribosomal peptide synthetase (NRPS) BGCs accounted for the highest proportion (22.9%, 8 clusters), followed by terpene and terpene-precursor BGCs (20.0%, 7 clusters combined). RiPP-like (8.6%, 3 clusters) and type I polyketide synthase (T1PKS) BGCs (8.6%, 3 clusters) were also identified (Table 3).

**Figure 7:**
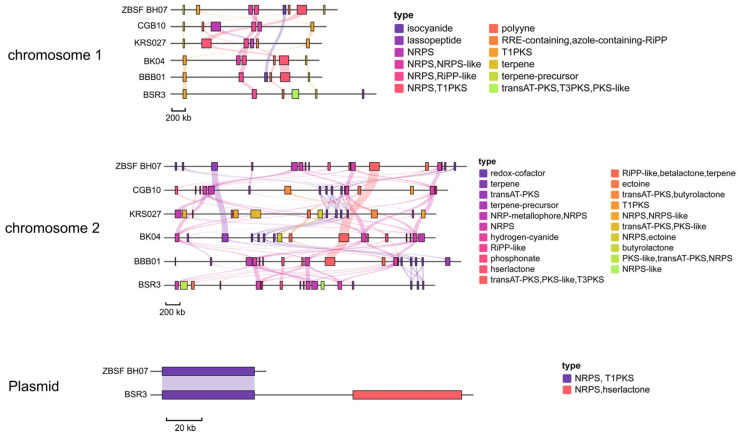
Predicted positions of biosynthetic gene clusters (BGCs) in the genome of *Burkholderia gladioli* ZBSF BH07 and other *B. gladioli* as identified by antiSMASH 8.0. The transparent lines represent gene clusters with homologous relationships between different strains.

A systematic comparative analysis of secondary metabolite BGCs was performed between ZBSF BH07 and its five reference strains (BK04, BBB01, CGB10, KRS027, and BSR3). The results revealed that all six strains harbored primarily NRPS and T1PKS BGCs, with terpene and trans-AT polyketide synthases (transAT-PKSs) as secondary types. Notably, while 10 BGCs were conserved across all strains, significant diversity in BGC types was observed among different strains. Additionally, Cluster 5 and Cluster 13 on chromosome 2—related to plantaribactin and barbamide biosynthesis—were shared among all six *B. gladioli* strains. Cluster 7 on chromosome 1, associated with orfamide B (a cyclic depsipeptide), was strain-specific and exclusively detected in ZBSF BH07, providing critical targets for future structural and functional studies of its products (Figure 7). Notably, genes encoding the biosynthesis of glidopeptin, syringomycin, gladiochelin A1/gladiochelin B2, icosalide A/icosalide B, and other metabolites were not identified in ZBSF BH07 (Figure 7 and Figure S4, Table S5). Moreover, the unique BGCs (transAT-PKS, T3PKS, and PKS-like) on chromosome 1 of BSR3, which are involved in the production of isobongkrekic acid/bongkrekic acid—a major toxic metabolite causing food poisoning—were characterized (Table S5).

## 4. Discussion

*Burkholderia* species represent a group of Gram-negative bacteria that are widely distributed in natural environments and are characterized by diverse metabolic capabilities and ecological adaptability. Certain members of this genus exhibit significant potential for biocontrol and plant growth promotion, highlighting their promising applications in sustainable agriculture. In this study, strain ZBSF BH07 was isolated from the rhizosphere soil of healthy grapevines. Phylogenetic analysis of the 16S rRNA, *gyrB*, *atpD*, *trpB*, and *rpoD* gene sequences identified it as *B. gladioli*. While *B. gladioli* has been reported to be pathogenic to both plants and humans [10,11,12,13], recent studies have demonstrated that certain strains show considerable potential in agriculture, exhibiting notable antifungal activity and growth-promoting effects in economically important crops such as cotton, rice, and sugarcane [16,26,44]. Our findings revealed that *B. gladioli* ZBSF BH07 displays potent antagonistic activity against 14 grapevine fungal pathogens, including *C. vitis*, *B. cinerea*, *B. dothidea*, and *Colletotrichum* spp. In controlling grape white rot, ZBSF BH07 demonstrated superior preventive efficacy: preventive treatment reduced lesion diameter by 31.1% (1.15 mm vs. 1.67 mm in curative treatment) and increased control efficacy by 21.86%. Growth promotion assays revealed that ZBSF BH07 significantly enhances grape seedling development, particularly by increasing root biomass. Compared with those of the untreated controls, the fresh and dry root weights increased by 172.9% and 231.34%, respectively. This robust root system not only facilitates improved nutrient and water uptake for grapevines but also contributes to soil stabilization and carbon sequestration, aligning with sustainable agriculture’s goal of enhancing soil health while boosting productivity.

We sequenced the complete genome of *Burkholderia gladioli* ZBSF BH07 and compared its genomic features with those of five beneficial strains (*B. gladioli* CGB10, KRS027, BK04, and BBB01) isolated from plants and one pathogenic strain (*B. gladioli* BSR3) (Table 1). The most pronounced differences were observed in terms of genome size and plasmid number. Strain BSR3 contained four plasmids, whereas *B. gladioli* BK04 harbored none. The genome sizes ranged from 9.05 Mb in BSR3 to 8.04 Mb in BK04. The mean GC content across all six strains was 68.12%, with individual strains deviating by no more than 0.72% from this average, with ZBSF BH07 exhibiting lower values.

ANI and DDH served as robust metrics for phylogenetic analysis at the genomic level. Strains are considered conspecific when they exhibit ANI > 96% and DDH ≥ 70% [39]. ANI/DDH analysis revealed that ZBSF BH07 had the highest similarity to strain CGB10, with values of 99.27% and 93.7%, respectively. Genomic collinearity (synteny) analysis evaluates structural conservation and gene order similarity across species or strains. This method also identifies conserved genomic regions and strain-specific insertions/deletions, providing insights into biological characteristics and potential applications. Using Mauve, we compared collinearity between ZBSF BH07 and strains CGB10, KRS027, BK04, BBB01, and BSR3. The results revealed high collinearity between ZBSF BH07 and CGB10, corroborating the ANI/DDH findings and supporting the previously established phylogenetic relationships. Gene family analysis elucidates functional diversity and evolutionary history. Comparative analysis of gene families in ZBSF BH07 versus related strains identified both shared and unique gene families.

PGPRs have garnered significant attention because of their ability to solubilize mineral phosphates, fix atmospheric nitrogen, and synthesize auxins such as IAA. These strains can also reduce host ethylene levels through 1-aminocyclopropane-1-carboxylate (ACC) deaminase activity, thereby increasing plant tolerance to abiotic stresses [45]. The results of the pot experiments in this study confirmed that the ZBSF BH07 strain significantly increased the grape seedling biomass. As a key regulator of multiple biological processes, IAA plays a critical role in plant growth and development. KEGG database analysis revealed genes associated with tryptophan biosynthesis (*trpA*, *trpB*, *trpC*, *trpD*, *trpE*, and *trpS*) and genes encoding key enzymes in IAA biosynthesis pathways within strain ZBSF BH07 (Table 2). These findings substantiate the mechanism by which ZBSF BH07 promotes grape growth through phytohormone regulation and demonstrate its potential for enhancing plant growth. This conclusion aligns with the observed significant stimulation of root development.

Another strategy by which PGPR enhances plant growth is phosphate solubilization. In soil, only 0.1% of phosphorus exists in soluble form available for plant uptake, due to its high fixation capacity and low solubility. To address this, phosphate-solubilizing bacteria in the rhizosphere secrete strong organic acids that dissolve insoluble phosphates, thereby increasing soluble phosphorus levels for plant absorption [46,47]. *Burkholderia* species are well-known for this trait; for example, *Burkholderia cepacia* can solubilize over 300 μg/mL of phosphate from insoluble Ca_3_(PO_4_)_2_ [21]. The primary mechanism underlying mineral phosphate dissolution involves the production of gluconic acid, mediated by the genes gcd (encoding glucose-1-dehydrogenase) and gad (encoding gluconate dehydrogenase) [48]. Notably, in strain ZBSF BH07, only the gcd gene was detected—suggesting it may still produce gluconic acid via a truncated pathway, albeit potentially with reduced efficiency. Once insoluble phosphates are solubilized into inorganic phosphate (Pi, PO_4_^3−^), both plants and bacteria require specific transport systems for uptake. In bacteria, this process is mediated by the phosphate-specific transport (PST) system, encoded by the *pstABCS* operon, which facilitates Pi import into the cell [49]. The pst operon comprises *pstS*, *pstC*, *pstA*, *pstB1*, and *pstB2*. *PstC* and *PstA* are integral inner membrane proteins, whereas *PstB* in *Escherichia coli* (*PstB1* and *PstB2* in *Bacillus subtilis*) is an ATP-binding protein [49,50]. Genomic analysis revealed that strain ZBSF BH07 carries the pst operon (*pstC*, *pstA*, *pstB*, and *pstS*), indicating its capacity for inorganic phosphate uptake from the environment, which is crucial for bacterial growth and metabolism. Another abundant source of phosphorus in soil is phosphonates. The *phn* gene cluster enables bacterial degradation of phosphonates, thereby releasing bioavailable phosphate for nearby plants [51]. Comparative genomic analysis demonstrated that ZBSF BH07 possesses *phnATSVUX*, a key set of phosphonate degradation genes. Pyrroloquinoline quinone (PQQ), a cofactor of glucose dehydrogenase (GDH), is involved in crucial glucose conversion processes [52]. The biosynthesis of PQQ involves six associated genes (*pqqABCDEF*), with pqqE being particularly important. Strain ZBSF BH07 contains five PQQ-related genes (*pqqABCDE*). Phosphate solubilization plate assays further confirmed that ZBSF BH07 can degrade both organic and inorganic phosphorus. Notably, its solubilization halo reached 2.8 cm during organic phosphorus degradation. Collectively, these results indicate that strain ZBSF BH07 can not only utilize inorganic phosphate but also transform complex organic phosphorus sources (such as phosphonates) into plant-available forms. This dual phosphorus acquisition strategy enhances the competitive fitness of ZBSF BH07 in phosphorus-limited environments and suggests its potential role in promoting soil phosphorus cycling and plant nutrition.

However, the physiological implications of IAA synthesis and phosphate solubilization under field conditions require further clarification. Pot experiments in this study were conducted under controlled environments with uniform soil matrices and microbial communities, which differ from the complex rhizosphere ecosystems of vineyards. Factors such as soil type, indigenous microbial competition, and seasonal climate fluctuations may modulate the efficacy of ZBSF BH07 in promoting plant growth. Future studies should validate these findings through field trials, focusing on strain persistence, interaction with native microbiota, and long-term impacts on soil nutrient cycling to bridge the gap between laboratory results and agricultural application.

Nitrogen fixation is the process by which microorganisms convert atmospheric nitrogen gas (N_2_) into ammonia or ammonium salts that are usable by plants. Previous studies have confirmed the presence of the *nifHDK* gene cluster within the *Burkholderia* genus, and nitrogen fixation has been demonstrated in several other *Burkholderia* strains [53,54,55]. Analysis of the KEGG database revealed that no nitrogen fixation genes were detected in the genome of strain ZBSF BH07. These findings indicate that strain ZBSF BH07 may lack the capacity to directly promote plant growth through nitrogen fixation. Nonetheless, this does not preclude the strain’s potential for plant growth promotion, as it may utilize other mechanisms, such as the production of the plant hormone IAA or phosphate solubilization. Furthermore, ZBSF BH07 may exhibit synergistic interactions with nitrogen-fixing microorganisms, collectively enhancing plant growth and development. Therefore, future research should further explore the plant growth-promoting mechanisms of strain ZBSF BH07 and its interactions with other microbes.

The antimicrobial activity of bacteria within the *Burkholderia* genus stems from their abundance of BGCs for secondary metabolites. Studies have shown that these bacteria can synthesize NRPSs, siderophores (such as pyochelin and pyoverdine), and phenazine compounds. These metabolites exert their antimicrobial effects by competitively chelating environmental iron ions or directly disrupting the cell membranes of pathogenic fungi. In this study, the complete genome (8.56 Mb) of strain ZBSF BH07 was found to contain 26 secondary metabolite BGCs. Notably, NRPS gene clusters were the most abundant, which aligns well with the broad-spectrum antimicrobial activity of the strain. As detailed in Table S5, among the identified clusters, 8 exhibited high similarity to known secondary metabolite clusters, 2 showed lower similarity, and 16 displayed no significant similarity to any known clusters.

In particular, strain ZBSF BH07 harbors a gene cluster encoding cyclic lipopeptide (CLP) orfamide B. Orfamide B is typically synthesized by *Pseudomonas* species via the NRPS pathway and possesses diverse biological activities, including antibacterial, antifungal, and insecticidal properties. This class of compounds, which is produced through complex biosynthetic pathways, serves as natural products widely applied in agricultural biocontrol. Their mechanisms of action include disrupting pathogen membrane integrity and inhibiting spore germination and infection structure formation. For example, orfamide B produced by *Pseudomonas* sp. CMR5c and CMR12a effectively reduced the incidence of rice blast by inhibiting appressorium formation and lysing spores in the pathogen. Furthermore, orfamide B exhibits direct lytic activity against zoospores of oomycetes such as *Phytophthora* and *Pythium* [56,57]. In the present study, microscopic observations revealed that ZBSF BH07 also caused lysis and rupture of hyphae from the pathogen *C. vitis* (Figure S1). This observation is consistent with the known antifungal activity of orfamide B, further confirming the potential of ZBSF BH07-produced orfamide B in biocontrol applications. However, the orfamide gene cluster in ZBSF BH07 has low similarity to known metabolite gene clusters, suggesting that the strain may have evolved unique biosynthetic pathways or regulatory mechanisms during its evolution. This uniqueness could be harnessed to develop novel biopesticides with reduced risk of pathogen resistance—a critical advantage over chemical fungicides, which often face rapid resistance development in target pests. Gene Cluster 5 on chromosome 2 shows high similarity to the siderophore plantaribactin produced by *B. plantarii*, a cluster commonly found and highly conserved across several analyzed strains. Plantaribactin-like siderophores not only function as iron transport vehicles but also serve as donors of the signaling molecule nitric oxide (NO) in plant roots, thereby contributing to both nutrient supply and plant health regulation. This dual functionality highlights their potential agricultural value [58]. The formation of a 2.5 cm orange–yellow halo by ZBSF BH07 on Chrome Azurol S (CAS) assay medium further confirmed its strong iron-chelating capacity.

Gene Cluster 5 on chromosome 1 is highly similar to sinapigladioside, whereas Gene Cluster 6 is highly similar to caryoynencin, both of which are produced by *Burkholderia gladioli*. Similarly, gene Cluster 12 on chromosome 2 is highly similar to lagriene, and gene Cluster 14 is highly similar to icosalide A/icosalide B, which are also metabolites of *B. gladioli*. These antimicrobial compounds protect vulnerable egg stages from harmful microorganisms [59,60,61]. They efficiently inhibit insect-pathogenic bacteria, such as Photorhabdus species, thereby protecting beetle larvae from infection and reinforcing the symbiotic defense system [62]. Furthermore, gladiostatin A, which was first isolated from *B. gladioli*, has significant inhibitory effects on multiple human cancer cell lines and inhibits tumor cell migration [63]. Gene Cluster 11 on chromosome 2 is highly similar to the nonribosomal peptide synthetase (NRPS)-derived secondary metabolite kolossin, which is produced by *Photorhabdus luminescens*. Naturally occurring kolossin A exhibits no significant inhibitory activity against common bacteria or fungi. It is speculated to function in interspecies communication, potentially by acting as a signaling molecule to regulate microbial community interactions [64].

Despite the significant biocontrol and plant growth-promoting potential of *Burkholderia* species, practical application requires rigorous assessment of safety risks associated with certain pathogenic strains. As previously noted, members of the *B. cepacia* complex pose opportunistic infection risks to humans, highlighting the necessity of genomic screening to eliminate virulence factor-carrying strains prior to agricultural use [65,66,67,68]. Notably, specific *B. gladioli* strains are also recognized pathogens, causing bulb rot in ornamental plants (e.g., gladiolus) and infections in immunocompromised individuals, further emphasizing the need for strain-specific safety validation [13]. In the case of ZBSF BH07, multiple lines of evidence support its low-risk profile: genomic analysis confirmed the absence of key toxin gene clusters (e.g., bongkrekic acid synthetase), while phenotypic assays demonstrated no hemolytic activity, phytotoxicity to grape tissues, or yellow pigment production (indicative of inactive toxoflavin pathways) (Figure 5A,B). Despite these reassuring findings, biosafety evaluations must extend beyond laboratory assays. Long-term persistence in agricultural ecosystems or horizontal gene transfer with indigenous soil microbiota could theoretically introduce unforeseen risks, such as the acquisition of virulence determinants. Thus, future field applications should incorporate monitoring of strain dissemination and impacts on non-target organisms to ensure environmental safety. Furthermore, even pre-screened “safe” strains require additional toxicological assessments and multi-season field trials to validate safety under real-world conditions.

## 5. Conclusions

*Burkholderia gladioli* ZBSF BH07 emerges as a dual-functional strain with significant potential for agricultural biocontrol and plant growth promotion. Its broad-spectrum antifungal activity (against 14 pathogens) and dual preventive/curative efficacy against grape white rot, combined with robust growth-promoting effects on grapevines, highlight its utility as a sustainable alternative to chemical inputs. Genomic analysis confirms the genetic basis for these traits, including abundant secondary metabolite biosynthesis gene clusters (notably NRPS) and conserved pathways for IAA production and phosphate solubilization, which collectively underpin its functional versatility. Safety evaluations further support its applicability, with no detected hemolytic activity or phytotoxicity to grapevines. These findings not only establish *B. gladioli* ZBSF BH07 as a promising candidate for grapevine disease management and growth enhancement but also provide a genomic framework for understanding the molecular mechanisms of beneficial Burkholderia–plant interactions. Ultimately, this work contributes to the development of eco-friendly agricultural strategies, aligning with global efforts to improve crop health and productivity while reducing environmental impact.

## Figures and Tables

**Table 1:** Genomic features of *Burkholderia gladioli* ZBSF BH07 and other *B. gladioli* strains.

Features	*B. gladioli* ZBSF BH07	*B. gladioli* CGB10	*B. gladioli* KRS027	*B. gladioli* BK04	*B. gladioli* BBB-01	*B. gladioli* BSR3
Biosample	SAMN48577572	SAMN15158960	SAMN34105703	SAMN28690464	SAMN17301201	SAMN02603164
Chromosome Number	2	2	2	2	2	2
Plasmid Number	1	1	1	0	1	4
Size (bp)	8,558,021	8,423,203	8,257,358	8,039,389	8,201,484	9,052,299
GC content (%)	68.06	68.13	68.67	68.24	68.19	67.40
Total genes	7431	7462	8844	6928	7090	7757
Predicted protein-coding genes	7262	6981	8781	6768	6941	7410
Ribosomal RNA	15	15	15	15	15	15
tRNA	68	67	66	67	66	68
CRISPR	25	6	/	/	6	/
Pseudogene	84	395	/	74	64	264
Origin	grape rhizosphere soil	sugarcane leaves	Cotton rhizosphere soil	Catalpa ovata rhizosphere soil	rice shoot	Rice
Reference	This study	[16]	[42]	[3]	[17]	[43]

**Table 2:** Genes involved in plant growth promotion in *Burkholderia gladioli* ZBSF BH07 and other *B. gladioli*.

Gene Name	Product	ZBSF BH07	CGB10		KRS027		BK04		BBB01		BSR3	
		ProteinID	ProteinID	Homology (%)	ProteinID	Homology (%)	ProteinID	Homology (%)	ProteinID	Homology (%)	ProteinID	Homology (%)
Phosphate solubilization genes												
*pstS*	phosphate ABC transporter substrate-binding	WP_013697368.1	WP_013697368.1	100.00	NA	99.66	WP_250804138.1	99.42	WP_013697368.1	100.00	WP_013697368.1	100.00
*pstC*	phosphate ABC transporter permease PstC	WP_013697369.1	WP_013697369.1	100.00	NA	100.00	WP_013697369.1	100.00	WP_013697369.1	100.00	WP_013697369.1	100
*pstA*	phosphate ABC transporter permease PstA	WP_105851495.1	WP_096749337.1	99.33	NA	99.66	WP_036034067.1	99.66	WP_036034067.1	99.66	WP_013697370.1	99.33
*pstB*	phosphate ABC transporter ATP-binding protein PstB	WP_013697371.1	WP_013697371.1	100.00	NA	100.00	WP_105826707.1	99.29	WP_013697371.1	100.00	WP_013697371.1	100.00
*phoU*	phosphate signaling complex protein PhoU	WP_013697372.1	WP_013697372.1	100.00	NA	100.00	WP_013697372.1	100.00	WP_013697372.1	100.00	WP_013697372.1	100.00
*phoB*	phosphate regulon transcriptional regulator PhoB	WP_025099650.1	WP_025099650.1	100.00	NA	100.00	WP_025099650.1	100.00	WP_025099650.1	100.00	WP_013697373.1	99.57
*phoR*	phosphate regulon sensor histidine kinase PhoR	WP_036052035.1	WP_036052035.1	100.00	NA	99.69	WP_036052035.1	100.00	WP_036052035.1	100.00	WP_013697374.1	99.54
	inorganic phosphate transporter	WP_017921247.1	WP_017921247.1	100.00	NA	99.70	WP_017921247.1	100.00	WP_017921247.1	100.00	WP_013698297.1	99.70
*gcd*	glucose/quinate/shikimate family membrane-bound PQQ-dependent dehydrogenase	WP_036056478.1	WP_096751037.1	99.88	NA	99.88	WP_036056478.1	100.00	WP_186039311.1	99.76	WP_013690155.1	99.76
*pqqA*	pyrroloquinoline quinone precursor peptide PqqA	WP_013691668.1	WP_013691668.1	100.00	NA	100.00	WP_013691668.1	100.00	WP_013691668.1	100.00	WP_013691668.1	100.00
*pqqB*	pyrroloquinoline quinone biosynthesis protein PqqB	WP_036028557.1	WP_046574788.1	99.34	NA	99.34	WP_013691667.1	99.67	WP_201446796.1	99.34	WP_013691667.1	99.67
*pqqC*	pyrroloquinoline-quinone synthase PqqC	WP_145756981.1	WP_145756981.1	100.00	NA	98.79	WP_250805862.1	99.20	WP_060001136.1	99.60	WP_043217028.1	98.80
*pqqD*	pyrroloquinoline quinone biosynthesis peptide chaperone PqqD	WP_025098424.1	WP_025098424.1	100.00	NA	100.00	WP_025098424.1	100.00	WP_025098424.1	100.00	WP_013691665.1	98.90
*pqqE*	pyrroloquinoline quinone biosynthesis protein PqqE	WP_186086773.1	WP_025098425.1	99.75	NA	99.25	WP_025098425.1	99.75	WP_105851184.1	99.25	WP_013691664.1	98.74
*phnX*	phosphonoacetaldehyde hydrolase	WP_431018100.1	WP_260865992.1	99.58	NA	99.07	WP_250805634.1	98.95	WP_186020012.1	98.60	WP_013691467.1	98.25
*phnA*	phosphonoacetate hydrolase	WP_431018012.1	WP_186036997.1	99.31	NA	98.71	WP_250804705.1	98.17	WP_201446705.1	98.17	WP_013689373.1	98.40
*phnV*	2-aminoethylphosphonate ABC transport system, membrane component PhnV	WP_017920137.1	WP_046582114.1	99.65	NA	99.30	WP_017920137.1	100.00	WP_036054457.1	99.65	WP_013689372.1	98.95
*phnU*	2-aminoethylphosphonate ABC transporter permease subunit	WP_186104004.1	WP_043217526.1	99.68	NA	99.15	WP_186078140.1	99.04	WP_043217526.1	99.68	WP_013689371.1	98.39
*phnT*	2-aminoethylphosphonate ABC transport system ATP-binding subunit PhnT	WP_036054458.1	WP_036054458.1	100.00	NA	100.00	WP_036054458.1	100.00	WP_036054458.1	100.00	WP_013689370.1	99.46
*phnS*	2-aminoethylphosphonate ABC transporter substrate-binding protein	WP_017920134.1	WP_017920134.1	100.00	NA	100.00	WP_250804704.1	99.72	WP_017920134.1	100.00	WP_013689369.1	99.17
Indole-3-acetic acid biosynthesis genes												
*trpC*	indole-3-glycerol phosphate synthase TrpC	WP_046581210.1	WP_096749797.1	98.85	NA	98.85	WP_096749797.1	98.85	WP_046581210.1	100.00	WP_013696524.1	98.47
*trpD*	anthranilate phosphoribosyltransferase	WP_025100550.1	WP_025100550.1	100.00	NA	99.42	WP_250803631.1	99.42	WP_025100550.1	100.00	WP_013696525.1	99.42
	anthranilate synthase component II	WP_013696526.1	WP_013696526.1	100.00	NA	100.00	WP_013696526.1	100.00	WP_025100549.1	99.49	WP_013696526.1	100.00
*trpE*	anthranilate synthase component I	WP_013696527.1	WP_013696527.1	100.00	NA	100.00	WP_013696527.1	100.00	WP_013696527.1	100.00	WP_013696527.1	100.00
*trpB*	tryptophan synthase subunit beta	WP_013690578.1	WP_013690578.1	100.00	NA	100.00	WP_186236507.1	99.75	WP_013690578.1	100.00	WP_013690578.1	100.00
*trpA*	tryptophan synthase subunit alpha	WP_046580979.1	WP_036055918.1	99.26	NA	99.26	WP_036055918.1	99.26	WP_201446989.1	98.89	WP_013690576.1	97.78
*trpS*	tryptophan--tRNA ligase	WP_046582086.1	WP_046582086.1	100.00	NA	99.44	WP_105857686.1	99.16	WP_046582086.1	100.00	WP_013689344.1	99.44
*iaaH*	indoleacetamide hydrolase	WP_160294375.1	WP_186271478.1	99.23	NA	NA	NA	NA	NA	NA	NA	NA
*aroC*	chorismate synthase	WP_036051822.1	WP_013697745.1	99.73	NA	99.18	WP_250804326.1	99.43	WP_013697745.1	99.43	WP_013697745.1	99.43
*aroA*	3-phosphoshikimate 1-carboxyvinyltransferase	WP_105859154.1	WP_036052403.1	99.31	NA	NA	WP_145757446.1	99.54	WP_036052403.1	99.31	WP_013696997.1	99.08
	shikimate kinase	WP_047837134.1	WP_080757405.1	99.46	NA	99.46	WP_047837134.1	100.00	WP_047837134.1	100.00	WP_047837134.1	100.00
*aroE*	shikimate dehydrogenase	WP_046576987.1	WP_186049681.1	99.32	NA	99.32	WP_059999883.1	99.66	WP_186049681.1	99.32	WP_013696600.1	97.60
*aroQ*	type II 3-dehydroquinate dehydratase	WP_013696594.1	WP_013696594.1	100.00	NA	100.00	WP_013696594.1	100.00	WP_013696594.1	100.00	WP_013696594.1	100.00
*aroB*	3-dehydroquinate synthase	WP_017921100.1	WP_166915270.1	99.72	NA	99.72	WP_250803588.1	99.72	WP_017921100.1	100.00	WP_013696404.1	99.72
*aroG*	3-deoxy-7-phosphoheptulonate synthase AroG	WP_013696710.1	WP_036048186.1	100.00	NA	99.72	WP_036048186.1	100.00	WP_105829091.1	99.72	WP_013696710.1	100.00

NA, not available.

**Table 3:** Information on Secondary Metabolites Predicted by AntiSMASH for *Burkholderia gladioli* ZBSF BH07.

Cluster	Type	From	To	Size (bp)	Most Similar Known Cluster	Similarity Confidence
Chromosome 1						
Cluster1	terpene-precursor	541,153	562,151	20,998		
Cluster2	T1PKS	741,213	788,865	47,652		
Cluster3	NRPS-like, NRPS	1,524,680	1,579,818	55,138	sulfazecin	High
Cluster4	RiPP-like, NRPS	1,606,244	1,670,249	64,005		
Cluster5	isocyanide	2,037,150	2,078,985	41,835	sinapigladioside	High
Cluster6	polyyne	2,114,617	2,141,003	26,386	caryoynencin	High
Cluster7	NRPS, T1PKS	2,248,353	2,388,098	139,745	orfamide B	Low
Cluster8	terpene	2,644,052	2,664,891	20,839		
Chromosome 2						
Cluster1	redox-cofactor	37,427	59,647	22,220		
Cluster2	terpene	138,663	159,733	21,070		
Cluster3	transAT-PKS	554,651	640,680	86,029	gladiostatinA	High
Cluster4	terpene-precursor	1,125,693	1,146,577	20,884		
Cluster5	NRP-metallophore, NRPS	1,693,445	1,777,734	84,289	plantaribactin	High
Cluster6	NRPS	1,798,931	1,855,874	56,943		
Cluster7	hydrogen-cyanide	1,887,604	1,900,630	13,026		
Cluster8	RiPP-like	1,939,225	1,950,241	11,016		
Cluster9	phosphonate	2,212,326	2,246,799	34,473		
Cluster10	hserlactone	2,510,928	2,531,542	20,614		
Cluster11	NRPS	2,539,442	2,602,838	63,396	kolossin	High
Cluster12	transAT-PKS, PKS-like, T3PKS	2,804,582	2,944,789	140,207	lagriene	High
Cluster13	terpene, betalactone, RiPP-like	3,602,604	3,644,478	41,874	barbamide	Low
Cluster14	NRPS	3,775,254	3,830,304	55,050	icosalide A/icosalide B	High
Cluster15	ectoine	3,831,482	3,841,868	10,386		
Cluster16	terpene	3,872,495	3,893,547	21,052		
Cluster17	terpene	4,008,965	4,032,489	23,524		
Plasmid						
Cluster1	NRPS, T1PKS	170,127	222,396	52,269		

## Data Availability

The original contributions presented in the study are included in the article/Supplementary Materials, further inquiries can be directed to the corresponding author.

## Supplementary Materials

The following supporting information can be downloaded at https://www.mdpi.com/article/10.3390/microorganisms13081756/s1, Figure S1: Antagonistic assay of *Burkholderia gladioli* ZBSF BH07. Mycelial growth (A) and mycelial morphology (C) on PDA medium; mycelial growth (B) and mycelial morphology (D) on the antagonistic assay plate. Figure S2: Phylogenetic tree for *Burkholderia gladioli* ZBSF BH07 and the genus *Burkholderia* based on 16S rRNA. The evolutionary history was inferred using the neighbor-joining method. The percentage of replicate trees in which the associated taxa clustered together in the bootstrap test (1000 replicates) is shown next to the branches. Figure S3: ANI (A) and DDH (B) value matrix heatmap between *B. gladioli* ZBSF BH07 and other *B. gladioli* strains; Figure S4: Comparison of antibiotic synthesis clusters of *B. gladioli* strains. Antibiotic synthesis clusters were identified using antiSMASH; Table S1: Information on strains and five housekeeping genes used for phylogenetic tree construction in this study; Table S2: Statistics of the genome assembly of *Burkholderia gladioli* ZBSF BH07; Table S3: Genome statistics of *Burkholderia gladioli* ZBSF BH07; Table S4: Genes associated with chemotaxis and assembly of flagella in *Burkholderia gladioli* ZBSF BH07 and other *B. gladioli* strains; Table S5: Secondary metabolite cluster identified in *Burkholderia gladioli* ZBSF BH07 and other *B. gladioli* strains.
