# Supplementary material for: Complete Genome Analysis and Antimicrobial Mechanism of Burkholderia gladioli ZBSF BH07 Reveal Its Dual Role in the Biocontrol of Grapevine Diseases and Growth Promotion in Grapevines
Source: Microorganisms. 2025 Jul 28;13(8):1756. doi: 10.3390/microorganisms13081756 (PMC12388634; doi:10.3390/microorganisms13081756)
Supplement: Supplementary file 1 [file microorganisms-13-01756-s001.zip › Table S2.pdf]

Table S2 Statistics of the genome assembly of *Burkholderia gladioli* ZBSF BH07.

Attribute	Value
Scaffold Length (bp)	8558021
Scaffold Number	3
Scaffold N50 (bp)	4049529
Scaffold N90 (bp)	4049529
Contig Length (bp)	8558021
Contig Number	3
Contig N50 (bp)	4049529
Contig N90 (bp)	4049529
GC content (%)	68.06
Gaps Number	0
