# Supplementary material for: Complete Genome Analysis and Antimicrobial Mechanism of Burkholderia gladioli ZBSF BH07 Reveal Its Dual Role in the Biocontrol of Grapevine Diseases and Growth Promotion in Grapevines
Source: Microorganisms. 2025 Jul 28;13(8):1756. doi: 10.3390/microorganisms13081756 (PMC12388634; doi:10.3390/microorganisms13081756)
Supplement: Supplementary file 1 [file microorganisms-13-01756-s001.zip › Table S3.pdf]

Table S3 Genome statistics of *Burkholderia gladioli* ZBSF BH07

Attribute	Value	% of total
Genome size (bp)	8558021	100.00
DNA coding (bp)	7437015	86.90
DNA G + C (bp)	5824589	68.06
Total repetitive sequence length (bp)	164741	1.92
Genomics Islands	306526	3.58
Total genes	7431	100.00
Protein coding genes	7262	97.73
Pseudo genes	84	1.13
Genes assigned to KEGGs	3402	45.78
Genes assigned to SwissProt	4254	57.25
Genes assigned to eggNOG	5835	78.52
Genes assigned to GOs	5484	73.80
Genes assigned to Pfam	6234	83.89
Genes assigned to NR	7134	96.00
Genes assigned to TCDB	2077	27.95
Genes assigned to PHI	2727	36.70
Genes assigned to CAZY	236	3.18
Genes with signal peptides	817	10.99
Genes with transmembrane helices	1657	22.30
Genes with Secreted Protein	589	7.93
Genes assigned to TrEMBL	7106	95.63
Genes assigned to CARD	9	0.12
Transmembrane protein	1657	22.30
Singal peptide	817	10.99
GIs number	17	N/D
Prophage_Num	3	N/D
CRISPR_Num	25	N/D

[a]N/D = not determined
