# Supplementary figures and images for: Complete Genome Analysis and Antimicrobial Mechanism of Burkholderia gladioli ZBSF BH07 Reveal Its Dual Role in the Biocontrol of Grapevine Diseases and Growth Promotion in Grapevines
Source: Microorganisms. 2025 Jul 28;13(8):1756. doi: 10.3390/microorganisms13081756 (PMC12388634; doi:10.3390/microorganisms13081756)

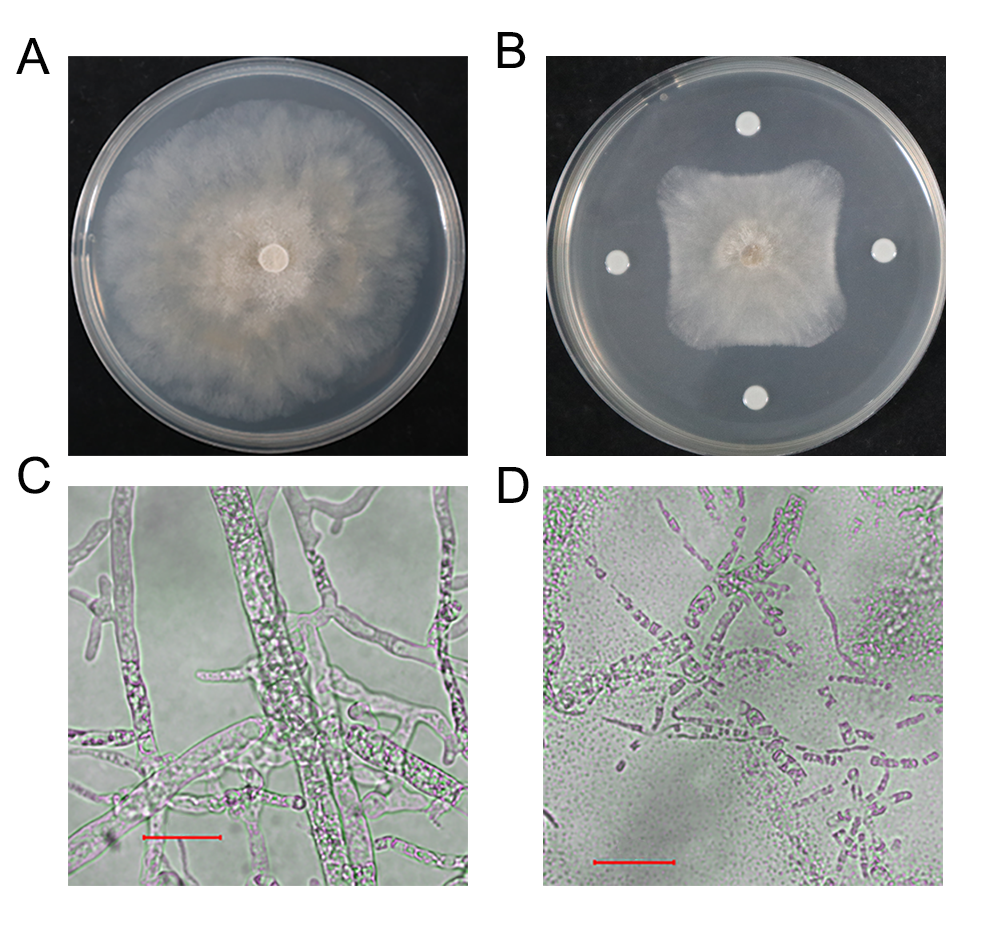

Supplement: Supplementary file 1 [file microorganisms-13-01756-s001.zip › Figure S1.png]

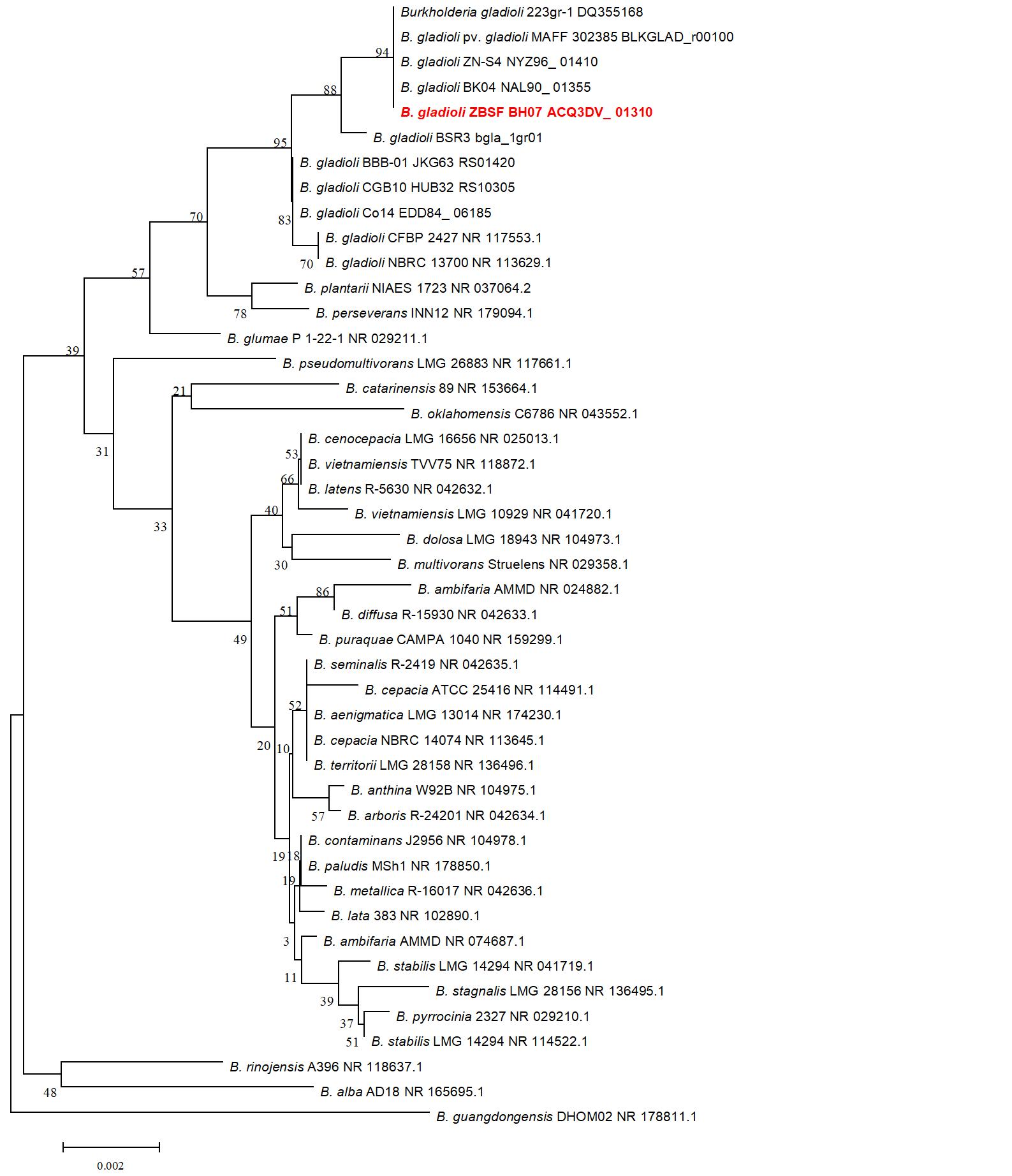

Supplement: Supplementary file 1 [file microorganisms-13-01756-s001.zip › Figure S2.jpg]

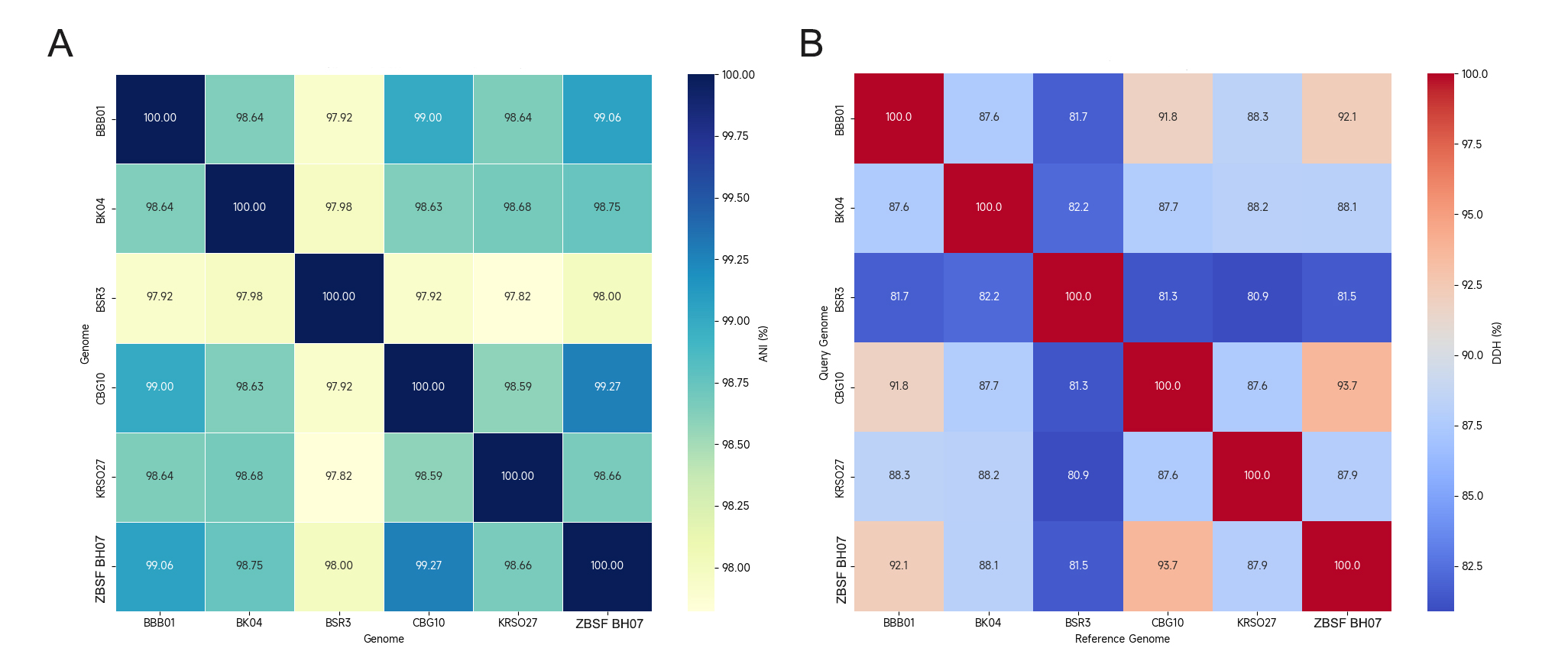

Supplement: Supplementary file 1 [file microorganisms-13-01756-s001.zip › Figure S3.jpg]

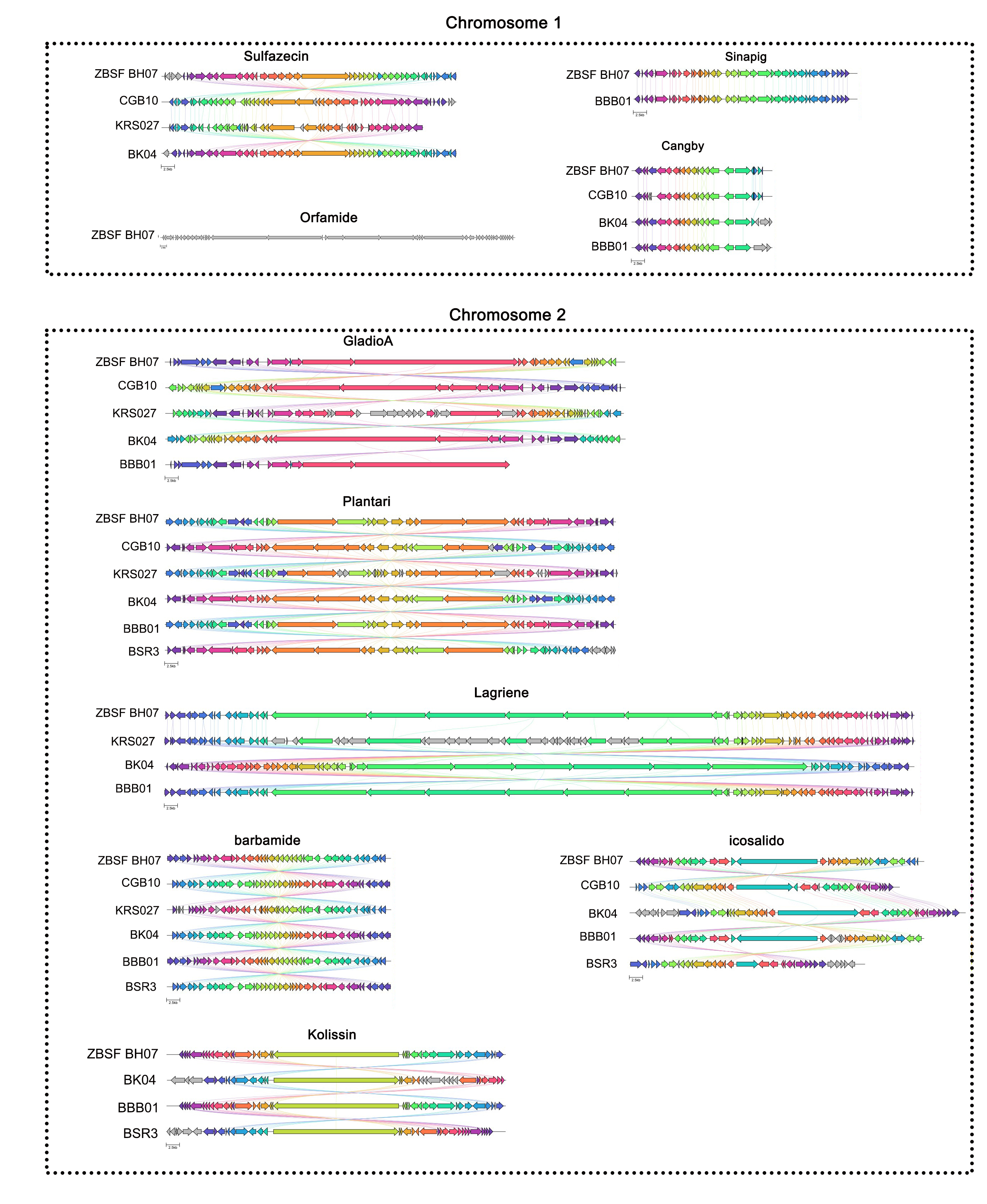

Supplement: Supplementary file 1 [file microorganisms-13-01756-s001.zip › Figure S4.jpg]
